# Whole-Genome Sequences of Propionibacterium australiense NML (LCDC) 98A072^T^ and NML (LCDC) 98A078, Associated with Granulomatous Bovine Lesions

**DOI:** 10.1128/MRA.01445-18

**Published:** 2018-11-21

**Authors:** Anne-Marie Bernier, Kathryn Bernard

**Affiliations:** a Department of Biology, Université de Saint-Boniface, Winnipeg, Canada; b National Microbiology Laboratory (NML)–CSCHAH Site, Public Health Agency of Canada, Winnipeg, Canada; c Department of Medical Microbiology, University of Manitoba, Winnipeg, Canada; Georgia Institute of Technology

## Abstract

Draft genome sequences of Propionibacterium australiense isolates NML 98A072^T^ and NML 98A078, derived from granulomatous lesions of infected bovines, were assembled and studied. Respectively, the genome sizes were 2.99 and 3.01 Mb, with G+C contents of 68.4% and 68.5%.

## ANNOUNCEMENT

In 2000, Forbes-Faulkner et al. described a novel granulomatous infection in cattle that was associated with an unidentifiable Propionibacterium*-*like organism (1). Propionibacterium australiense sp. nov. was subsequently described as this novel bovine pathogen (2). Here, we have characterized draft genome sequences for two P. australiense strains, NML/LCDC 98A072^T^ (= ATCC BAA-264^T^ = CCUG 46075^T^) and NML/LCDC 98A078 (= ATCC BAA-263 = CCUG 46174). Identifiers using NML (National Microbiology Laboratory) or the older acronym LCDC (Laboratory Centre for Disease Control) are to be considered synonyms. Bacteria were subcultured after storage at −80°C in Microbank vials (Pro-Lab) from NML stocks and passed twice at 35°C on Brucella blood agar plates (BBA; Thermo Fisher) for 48 h under anaerobic conditions in a jar containing a GasPak (BD).

A loopful of plate culture was passed and grown anaerobically in prereduced peptone-yeast extract broth (Anaerobe Systems) for 18 h at 35°C. DNA was extracted using a DNA minikit (Qiagen), and paired-end whole-genome shotgun libraries were constructed using a Nextera XT library preparation kit. Samples were run separately for sequencing on the MiSeq 600-cycle kit (version 3) on a MiSeq sequencer (Illumina). Read quality was assessed with FastQC (http://www.bioinformatics.babraham.ac.uk/projects/fastqc/) and assembled using default settings of SPAdes (version 3.9.0 [3]) after merging short paired-end reads with Fast-Length Adjustment of Short Reads (FLASH) with default settings (4).

The genomes were compared to each other using JSpeciesWS to calculate the average nucleotide identity values using BLAST+ (ANIb) (5). With that approach, 98A072^T^ and 98A078 had ANIb scores more than 99.6% similar to each other but only 73.56% to 84.11% similar to genomes from Propionibacterium freudenreichii subsp. freudenreichii DSM 20271^T^ (GenBank accession number CP010341) and Propionibacterium acidifaciens DSM 21887^T^ (GenBank accession number AUFR00000000), species selected from the genus Propionibacterium after emendation in 2016 (6). The genome-to-genome distance calculator (7) was used to estimate *in silico* DNA-DNA hybridization values between strains. The two NML strains were found to have 97.6% similarity to each other using the recommended formula 2 for draft genomes, but values were low (∼20%) when compared to those of P. freudenreichii subsp. freudenreichii DSM 20271^T^.

The sequencing run for NML 98A072 produced 462,927 sequences of 35 to 301 bases in length, with a total of 136,192,279 bases. The draft genome of NML98A072^T^ was comprised of 2,996,433 bp, which assembled into 63 contigs with 82× coverage, a G+C content of 68.4%, and an *N*_50_ contig length of 138,977. The genome, annotated by Prokka (version 1.13) (8), coded for 2,578 proteins, of which 85% were assigned to Clusters of Orthologous Groups (COG) categories using eggNOG-mapper (9). This genome encoded 3 rRNA genes, 51 tRNAs, and 9 repeat CRISPR elements. The sequencing run for NML 98A078 produced 408,707 sequencing reads (lengths of 35 to 301 bases), with a total of 113,773,758 bases. The draft genome of NML 98A078 consisted of 3,016,395 bp, assembled into 63 contigs with 64× coverage, a G+C content of 68.5%, and an *N*_50_ contig length of 129,689. eggNOG-mapper assigned 83.5% of the 2,644 coding regions to COG categories, and the draft genome encoded 3 rRNA genes, 51 tRNAs, and 4 CRISPR repeats. Neither genome harbored intact phages, as evaluated using PHASTER (10). The G+C contents of the draft genomes were consistent with members of the emended genus Propionibacterium (6, 11).

### Data availability.

Raw reads for these projects were assigned accession numbers SRX4875582 and SRX4875583 in the NCBI Sequence Read Archive. Draft genome sequences of Propionibacterium australiense 98A072^T^ and 98A078 were deposited in DDBJ/ENA/GenBank under the accession numbers RCIV00000000 and RCIW00000000, and the versions described in this paper are RCIV01000000 and RCIW01000000.
